# U6 can be used as a housekeeping gene for urinary sediment miRNA studies of IgA nephropathy

**DOI:** 10.1038/s41598-018-29297-7

**Published:** 2018-07-18

**Authors:** Zhi-Yu Duan, Guang-Yan Cai, Ji-Jun Li, Ru Bu, NanNan Wang, Pei Yin, Xiang-Mei Chen

**Affiliations:** 1 Department of Nephrology, Chinese PLA General Hospital, State Key Laboratory of Kidney Diseases, National Clinical Research Center for Kidney Diseases, Beijing, 100853 China; 2 Department of Nephrology, First Affiliated Hospital of Chinese PLA General Hospital, Beijing, 100048 China

## Abstract

Recent studies have indicated that urinary sediment miRNAs not only are able to serve as non-invasive diagnostic biomarkers for IgA nephropathy (IgAN) but may also be closely related to several clinical and pathological indicators. However, the lack of a suitable internal reference miRNA has hampered research into urinary sediment miRNAs. To date, U6 has been used as a reference gene in urinary sediment miRNA studies mostly based on the results from studies using tissue samples and cell lines. In a total of 330 IgAN patients, 164 disease control patients and 130 normal control patients, there was no significant difference in U6 levels. We also compared the U6 levels in different types of primary glomerular disease groups (IgA nephropathy, membranous nephropathy, minimal change nephrosis and focal segmental glomerular sclerosis). The results confirmed that there was no significant difference in the expression of U6 in different primary glomerular disease groups. Moreover, treatment had no significant effect on the expression levels of U6 in IgA nephropathy. Therefore, U6 is an excellent housekeeping gene for urinary sediment miRNA studies of IgA nephropathy.

## Introduction

IgA nephropathy (IgAN) is the most common primary glomerulonephritis in the world and is also one of the main causes of end-stage renal disease (ESRD) in China^1^. The majority of IgAN cases are progressive, and approximately 15–40% of patients will develop ESRD within 5–25 years after being diagnosed^2^. The diagnosis of IgAN relies entirely on a renal biopsy, which is invasive and cannot be frequently repeated whenever the illness warrants. Therefore, the development of non-invasive biomarkers will be of great significance for the clinical assessment of IgAN.

MicroRNAs (miRNAs) are a class of small non-coding RNAs that regulate gene expression at the post-transcriptional level^3^. Many studies, including our previous studies, found that miRNAs may have important roles in the pathogenesis and progression of IgA nephropathy^4–10^. Abnormal expression of miR-148b in peripheral blood mononuclear cells may account for the aberrant glycosylation of IgA1 observed in patients with IgA nephropathy^9^. Furthermore, there is a limited number of human miRNAs, with each individual miRNA modulating the protein output from hundreds of target genes^11^. Urinary sediment miRNAs directly originate when passing through the kidney tissue. Moreover, they have many clinical advantages, such as being non-invasive and easy to obtain. Urinary sediment miRNAs not only are able to serve as non-invasive diagnostic biomarkers for IgA nephropathy^7^ but may also be closely related to several clinical and pathological indicators^6,8^ that can predict therapeutic efficacy and disease progression. However, as the cornerstone of urinary sediment miRNA biomarkers, the reference gene (housekeeping gene) remains unknown.

U6 is a type of small nuclear RNA (snRNA) and is highly conserved among species^12^. U6 snRNA located at the heart of the spliceosome participates in the processing of mRNA precursors^13^. U6 is very stable because of the combination of small nuclear ribonucleoprotein complexes, a 5′ cap, a 3′U-rich tail, and the capacity for self-and/or U4 hybridization^14,15^. The half-life value is approximately 24 hours^14,16^. U6 is one of the most widely used internal reference genes for miRNA. U6 has been used as an internal reference gene in renal tissue^17^, cell lines^18^ and peripheral blood mononuclear cells^10^ in kidney disease patients. To date, U6 has been used as a reference gene in urinary sediment miRNAs studies^4–8,19^ mostly based on data from studies using tissue samples and cell lines^17,18^. However, no data are available concerning reference genes for urinary sediment miRNAs in IgAN patients, and a study with both healthy controls and disease controls is lacking.

In this study, we compared the difference in the expression levels of U6 between an IgAN group, disease control (DC) group and normal control (NC) group. We also compared the different expression levels of U6 in the IgAN group and disease control group before and after treatment. We found that the expression levels of U6 in urinary sediment in patients with IgAN were very stable. U6 could be suitable as an internal reference gene in the study of urinary sediment miRNAs.

## Results

### Patients characteristics

Demographic and clinical characteristic, provided in Table 1, were comparable between IgAN patients and controls in each group. There were no significant differences in age, sex distribution, serum creatinine (Scr) and estimated glomerular filtration rate (eGFR) among different groups. However, the 24-hour urinary protein excretion (UPE) and urinary N-acetyl glucosaminidase (NAG) were significantly lower in the IgAN group than the DC group (all P < 0.001). Serum albumin in the IgAN group was significantly higher than in the DC group.

**Table 1:** Demographic and baseline clinical data of all subjects.

	IgAN	DC	NC	P value
Gender (M)	177(53.64%)	84(51.22%)	68(52.31%)	0.875
Age (year)	34.64 ± 9.79	35.2 ± 10.39	35.2 ± 11.94	0.864
Albumin (g/L)	37.89 ± 5.9	27.5 ± 8.23	—	<0.001
UPE (g/day)	1.25 ± 1.28	3.31 ± 2.58	—	<0.001
Scr (μmol/L)	102.21 ± 86.84	100.31 ± 42.27	—	0.625
eGFR (ml/min/1.73 m^2^)	97.08 ± 67.08	93.83 ± 29.12	—	0.371
Urinary NAG	29.45 ± 21.62	59.12 ± 54.98	—	<0.001

DC, disease control; eGFR, estimated glomerular filtration rate; NC, normal control; NAG, N-acetyl glucosaminidase; Scr, serum creatinine; UPE, 24-hour urinary protein excretion.

### Confirmation study

**Figure 1:**
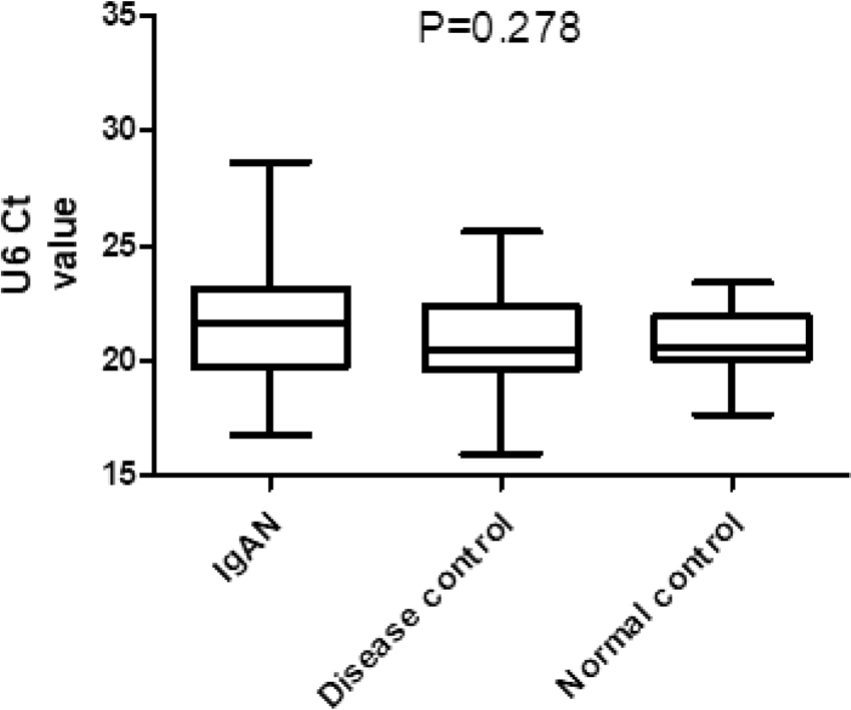
Comparison of urinary sediment U6 expression levels between the groups in a confirmation cohort.

In the confirmation cohort, the U6 levels were validated in 69 IgAN patients, 45 disease control patients and 32 normal control persons (Fig. 1). One-way analysis of variance (ANOVA) was used to check whether a significant difference in U6 values existed between those three groups. The P-value was 0.278 (IgAN 21.584 ± 2.565 vs DC 21.119 ± 2.261 vs NC 20.864 ± 1.306). The results showed that there was no significant difference in the expression levels of U6 in the IgAN group, disease control group and normal group.

### Validation study

**Figure 2:**
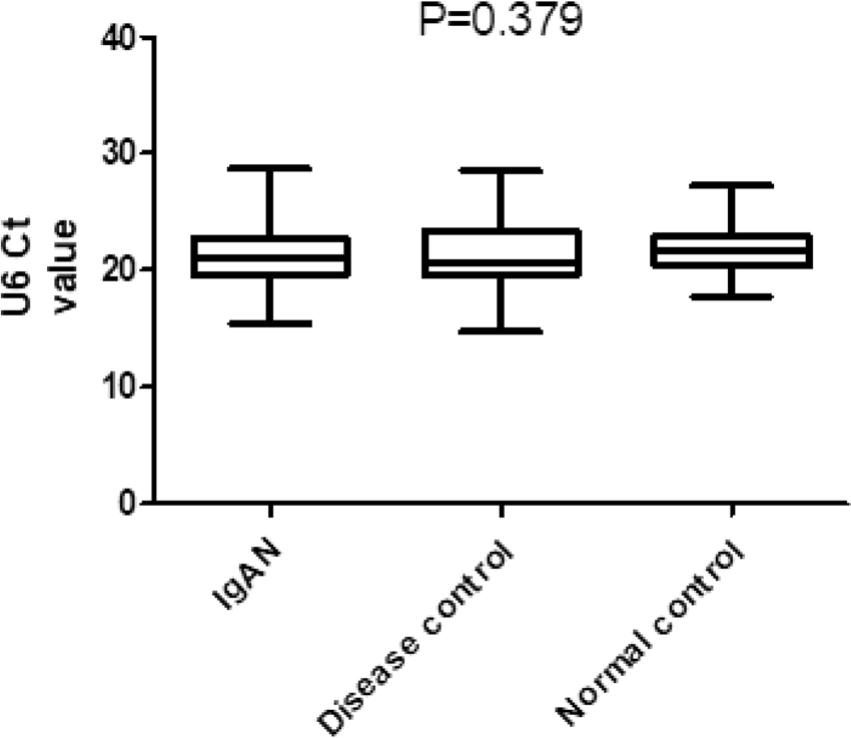
Comparison of urinary sediment U6 expression levels between the IgAN group and control group in a validation cohort.

The U6 levels were further validated in the validation cohort (Fig. 2). In the validation cohort, we expanded the samples to 330 IgAN patients, 164 DC patients and 130 NC persons. The results showed that the U6 levels were not significantly different between groups (IgAN 21.162 ± 2.529 vs DC 21.380 ± 2.651 vs NC 21.586 ± 2.002; P = 0.379; ANOVA). In the comparison between the two groups, there were also no significant difference between IgAN group and DC group (P = 0.377), IgAN group and NC group (P = 0.204), and DC group and NC group (P = 0.525). Two-tail Student’s t-test was used to compare U6 levels between the two groups. The baseline expression levels (before treatment) of U6 were fairly stable between the IgAN group and control group.

### Expression stability of U6 in the different primary glomerulonephritis groups

**Figure 3:**
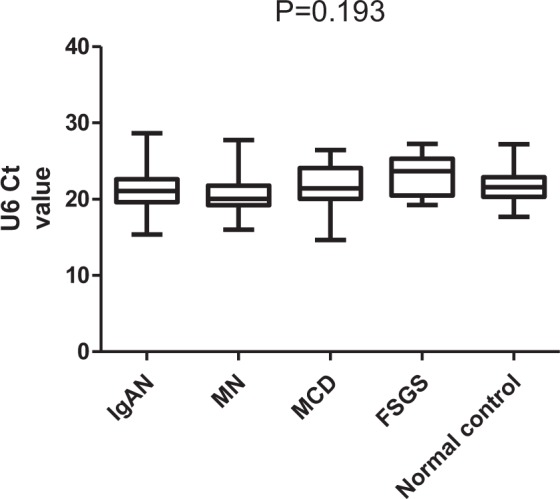
Comparison of urinary sediment U6 expression levels between the different primary glomerulopathy groups.

The results showed that there was no significant difference in the expression levels of U6 between the DC group, the IgAN group and the NC group. Therefore, we hypothesized that there was also no significant difference in the expression level of U6 between different glomerular diseases groups. To verify this hypothesis, the different expression levels of U6 were compared between the IgAN group, MN (membranous nephropathy) group, MCN (minimal change nephrosis) group and FSGS (focal segmental glomerular sclerosis) group. There was no significant difference in the expression levels of U6 in the different primary glomerular disease groups (IgAN 21.162 ± 2.529 vs MN 20.839 ± 2.575 vs MCN 21.698 ± 2.717 vs FSGS 21.72 ± 2.178; P = 0.231; ANOVA). There was no significant difference between the primary glomerular disease group and the NC group (P = 0.193) (Fig. 3). Therefore, U6 may also be used as the housekeeper gene in the primary glomerular disease group.

### The effect of treatment on the levels of U6 in the IgAN group

**Figure 4:**
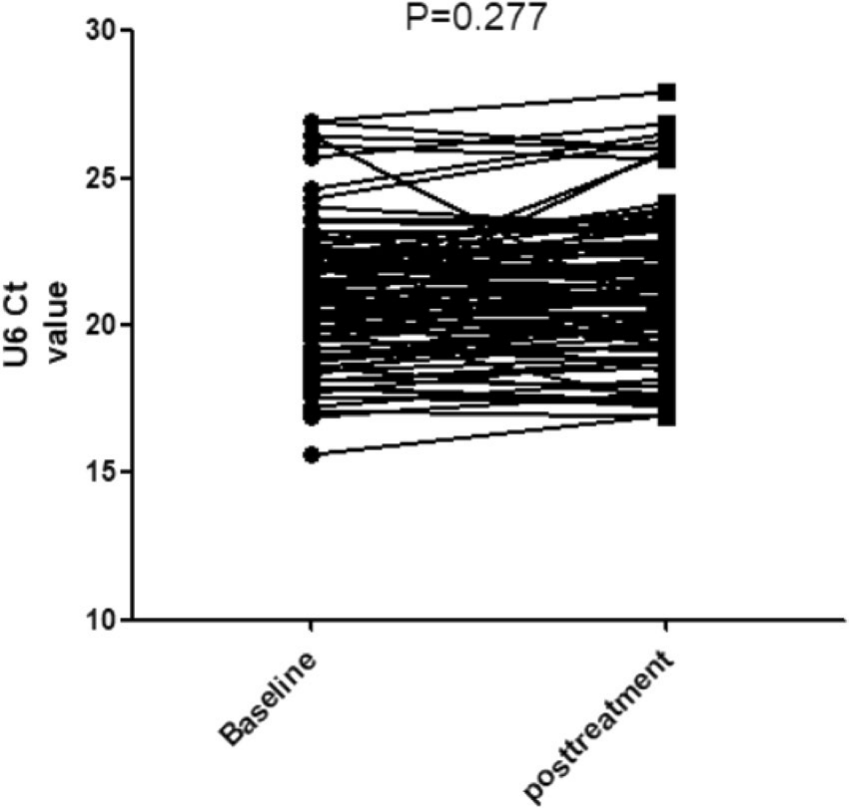
U6 values in IgAN group before and after treatment.

**Figure 5:**
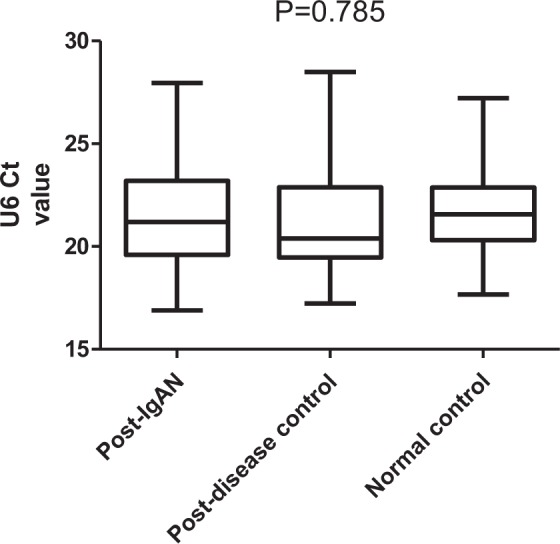
Comparison of urinary sediment U6 expression levels between the IgAN group, disease control group and normal control group after treatment.

To verify the effect of treatment on the U6 value of the IgAN group, 90 IgAN patients were retested at approximately 1 year (10.89 ± 2.93 months) after treatment. The results showed that there was no significant difference in the expression of U6 values before and after treatment in the IgAN group (baseline 21.022 ± 2.331 vs post-treatment 21.417 ± 2.526; P = 0.277; Two-tail Student’s t-test) (Fig. 4). We also compared the differences in U6 levels between the IgAN group, disease control group and healthy control group after treatment. The results (Fig. 5) also showed that there was no significant difference between the three groups (IgAN 21.417 ± 2.526 vs DC 21.273 ± 2.528 vs NC 21.586 ± 2 0.002; P = 0.785; ANOVA). Therefore, the treatment may have no significant effect on urinary sediment U6 values in IgA nephropathy.

### The effect of treatment on the levels of U6 in the primary glomerulopathy group

**Figure 6:**
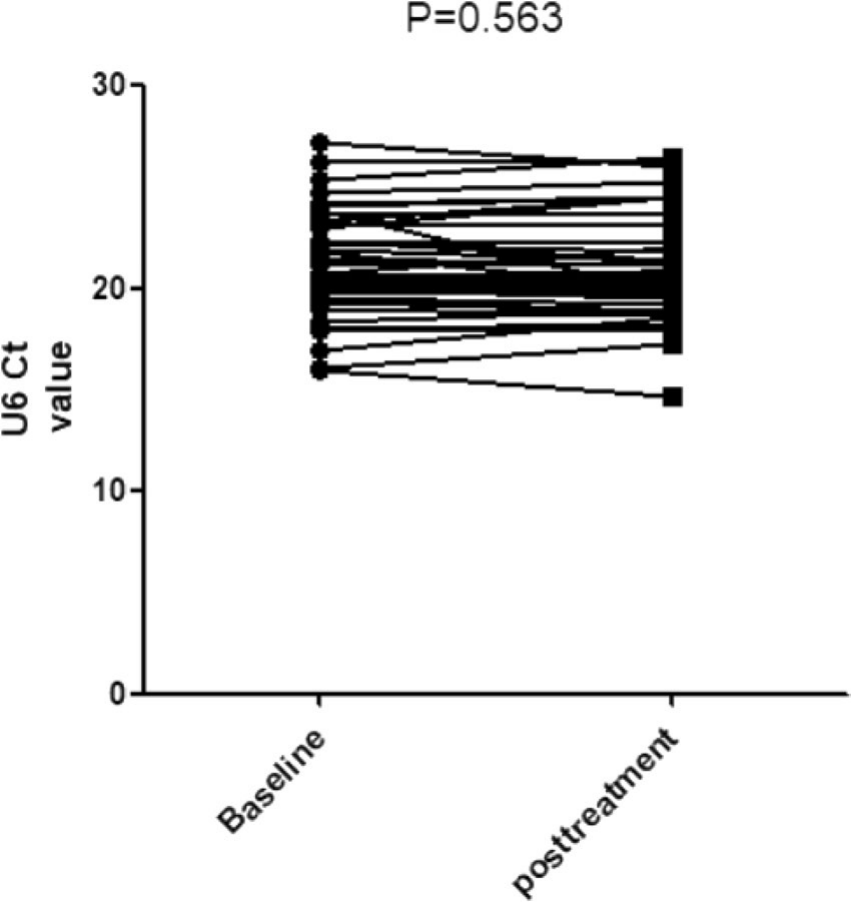
U6 values in the primary glomerulopathy group before and after treatment.

The retests were also performed on 46 disease control patients at approximately 1 year (10.89 ± 2.93 months) after treatment. Similar to those in the IgAN group, there were no significant differences in the expression of U6 levels before and after treatment (Fig. 6) in the DC group (baseline 20.970 ± 2.475 vs posttreatment 21.273 ± 2.528; P = 0.563; Two-tail Student’s t-test). Therefore, the treatment may have no significant effect on urinary sediment U6 values in primary glomerulonephritis.

## Discussion

Our recent work showed that urinary sediment miRNAs may play an important role in diagnosing IgAN and predicting therapeutic efficacy and disease progression^6–8^. Several miRNAs have been found to be useful non-invasive biomarkers of IgAN in our previous research^7^. However, the lack of a suitable internal reference gene (internal reference miRNA) has hampered research and the application of urinary sediment miRNAs.

U6 is an snRNA that was discovered by immunoprecipitation from mouse cell nuclear extract, along with U1, U2, U4, and U5 snRNAs^20^. U6 is highly conserved among species^12^ and relatively stable in different tissues and cells from the same organism. U6 is one of the most commonly used miRNA housekeeping genes and is often used in the study of IgAN kidney tissue and renal cell lines^18,21,22^. Currently, U6 is the most widely using reference gene in urinary sediment miRNAs studies^4–8,19,23^ that are primarily based on studies using tissue samples and cell lines^17,18^. However, there are many obvious differences between urinary sediment and renal tissue or renal cell lines. The most important is that there are too many factors of change between individuals and even within the same person, showing significantly different urinary sediment compositions at different time points. Different types of renal pathology, chronic kidney disease (CKD) staging, drugs, renal perfusion, exercise, diet and even body position all affect the type, quantity and proportion of urinary sediment cells. Therefore, directly applying the results derived from renal tissues and cell lines to urinary sediment research without verification is not advisable. To date, no data are available concerning reference genes of urinary sediment miRNAs in IgAN patients, and a study with both healthy controls and disease controls is lacking.

Here, we compared the difference in the expression levels of U6 in the IgAN group, disease control group and normal group. We found that the values of U6 were fairly stable between the IgAN group and control group. The results were consistent with the results of the renal tissue and renal cell line^17,18^. Moreover, in our previous study^7^, we detected the value of U6 in peripheral blood mononuclear cells, human primary tubular epithelial cells and red blood cells, and found no significant difference. Mononuclear cells, human primary renal tubular epithelial cells and red blood cells were the main cell types of urinary sediment both in the IgAN group, disease control group and normal group.

The disease control group in this study contained other glomerular diseases except IgAN, including primary glomerular disease, such as MN, FSGS, MCN, non-IgA mesangial proliferative glomerulonephritis, and secondary glomerular disease, such as Henoch-Schonlein purpura nephritis (HSPN) and renal amyloidosis. The results showed that there was no significant difference in the expression levels of U6 between the disease control group, the IgAN group and the NC group. Therefore, we proposed that there was also no significant difference in the expression levels of U6 between different glomerular disease groups. We compared the U6 levels in different types of primary glomerular disease groups (IgAN, MN, MCN and FSGS). The results confirmed that there was no significant difference in the expression of U6 in different primary glomerular disease groups. There was also no significant difference in the expression of U6 between the primary glomerular disease group and the NC group. However, we also observed that the sample size of the IgAN group was much larger than that of other primary glomerulonephritis as the disease control group, particularly the retest of patients after treatment; therefore, we could not exclude the possibility of bias. U6 may be a housekeeping gene for the primary glomerular disease group, but the a larger sample size is needed to validate these findings.

In addition to maintaining stability in different disease conditions, a good housekeeping gene should also be stable after different treatments. However, many studies including our previous studies found that some urinary sediment miRNAs were closely related to the treatment response and complete remission (CR) rate^4–6,8^. All patients in this study were not treated, and the samples were retained before renal biopsy. Whether U6 would be affected by different treatments was not known. To determine whether the expression levels of U6 were affected by different treatments, we resampled and retested urinary sediment from 90 IgAN patients and 46 other primary glomerular disease patients after approximately one year of treatment. The treatment plan for patients was formulated according to the Kidney Disease: Improving Global Outcomes (KDIGO) related guidelines. The expression levels of U6 in the IgAN group and the other primary glomerular disease group were not changed before and after treatment. Moreover, there were no significant differences in the U6 expression levels between the IgAN group, the other primary glomerular disease group and the NC group after treatment. The differences in the U6 mean values between the IgAN group and the other primary glomerular disease group before and after treatment were both less than 0.4 (original Ct value), which was far less than the value of its standard deviation. Therefore, the treatment had no significant effect on the expression levels of U6 in IgAN.

The study has some limitations. First, the sample size of the IgAN group was much larger than that of the other primary glomerulonephritis, particularly during the retest of patients after treatment; therefore, we cannot exclude the possibility of bias. However, in this preliminary study, we found that there was no significant difference in the expression of U6 in different primary glomerular disease groups. Further studies involving a larger sample are needed in the future. Second, although there was no difference in the expression levels of U6 before and after treatment, whether different treatments, such as angiotensin-converting enzyme inhibitors/angiotensin receptor blocker (ACEI/ARB), glucocorticoids and immunosuppressants, had different effects on the expression levels of U6 were undefined. Nevertheless, either the results of IgAN (ACEI/ARB as the main treatment), or the results of MN, MCN and FSGS (glucocorticoids and immunosuppressants as the main therapy), showed that there was no significant difference in the expression levels of U6 before and after treatment. We hypothesized that both immunosuppressive therapy and blocking the renin- angiotensin-aldosterone system (RAAS) may have no significant effect on the expression levels of U6. Further studies are necessary to confirm the effect of different treatments on the expression levels of U6.

In summary, this is the first study to investigate the reference gene of urinary sediment miRNAs in primary glomerular disease. The data presented here demonstrate that U6 can be used not only as a housekeeping gene in IgAN but also as a housekeeping gene in primary glomerulonephritis. The expression levels of U6 were fairly stable before and after treatment.

## Methods

### Sample collection

A total of 330 patients with biopsy-proven IgAN were included, and 130 participants from the normal control group were matched by sex and age. In addition, 164 patients of the DC group were also included: 90 patients with MN, 25 patients with FSGS, 37 patients with MCN, 5 patients with HSPN, 5 non-IgA mesangial proliferative glomerulonephritis and 2 patients with renal amyloidosis. The disease control group included in this study contained other glomerular diseases except IgAN, including primary glomerular disease and secondary glomerular disease. After the renal biopsy, the patients were treated with corticosteroids or ACEI/ARB, according to the KDIGO guidelines. The study was performed in accordance with the Declaration of Helsinki for Human Research and approved by the Ethics Committee of the Chinese PLA General Hospital. Written informed consent for inclusion was obtained from each participant. The inclusion criteria were as follows: patients signed the informed consent and were age ≥18 years. We excluded the following: patients who received corticosteroids or immunosuppressants before the beginning of this study; patients who had undergone kidney transplantation and who were undergoing dialysis; the number of glomerulus in renal biopsy tissues ≤8; or patients who were pregnant, planning a pregnancy, or those who were breastfeeding.

The demographic and clinical data, such as age, gender, albumin, Scr, urinary NAG and 24-hour UPE, of all included participants were recorded at the time of kidney biopsy. The eGFR was estimated with the Asian modified CKD-EPI equation^24^.

### Sample preparation

Whole stream early morning urine specimens were collected from patients and controls on the day of renal biopsy. The urine sample was processed within 4 hours after collection at 4 °C. Each urine sample was centrifuged at 3000 g for 30 minutes and 13000 g for 15 minutes at 4 °C^4^. The urine supernatant was stored at −80 °C until use.

### RNA extraction

TRIzol (Invitrogen, USA) was used for the extraction of total RNA from urinary sediment according to the manufacturer’s protocol. When the total RNA level of the sample was less than 50 ng/μl, we discarded this sample, because the sample may be degraded. The quantity and purity (ratio of absorbances of the RNA isolates at 260 nm and 280 nm [A260/280]) of the RNAs obtained was evaluated with a NanoDrop 2000 spectrophotometer (Thermo Scientific, Waltham, Massachusetts, USA). The ratio of A260/280 for all samples was between 1.8–2.2. Samples less than 1.8 were considered contaminated by organic substances, such as protein. Additionally, samples greater than 2.2 may be considered hydrolyzed. Both situations were identified as failure of sample extraction, and samples were again retained.

### RT-qPCR analysis

We used the miRcute miRNA First-Strand cDNA Synthesis Kit (Tiangen Biotech, Beijing, China) and miRcute miRNA qPCR Detection kit (Tiangen Biotech, Beijing, China) for reverse transcription and quantitative detection. Urinary sediment hsa-U6 (Applied Biosystems) was quantified by real-time quantitative polymerase chain reaction (RT-QPCR) using the ABI Prism 7500 Sequence Detection System (Applied Biosystems, Foster City, CA, USA). For RT-PCR, the dissolution curves were single peaks and excluded the results of multimodal peaks. All reactions were run in triplicate. The value of U6 is represented by the measured original Ct value.

### Statistical analyses

Statistical analysis and graphing were performed with the SPSS 24.0 and GraphPad Prism 5.01 for Windows (GraphPad Software Inc., San Diego, CA, USA). U6 levels were expressed as the mean ± standard deviation (SD). Two-tail Student’s t-test was used to compare U6 levels between two groups where appropriate. Nonparametric Mann–Whitney or Kruskal–Wallis rank tests were used for testing the parameters of data that were not normally distributed. One-way analysis of variance (ANOVA) followed by the Student-Newman-Keuls posttest was used to determine whether a significant difference in U6 values existed between multiple groups. P < 0.05 was considered statistically significant. All probabilities were two-tailed.
